# Physiological Impact of Environmental Stressors During Neonatal Transport: A Systematic Review of Clinical and Biophysical Outcomes

**DOI:** 10.7759/cureus.111621

**Published:** 2026-06-27

**Authors:** Hana Damra, Abubakr Elmahdi, Nesreen Bushara

**Affiliations:** 1 Neonatology, University Hospitals of Leicester NHS Trust, Leicester, GBR; 2 Paediatrics, Glan Clwyd Hospital, Bodelwyddan, GBR; 3 Paediatrics, University Hospitals of Morecambe Bay NHS Foundation Trust, Cumbria, GBR

**Keywords:** ambulance, intraventricular haemorrhage, neonatal transport, noise, physiological stability, systematic review, thermoregulation, vibration

## Abstract

Neonatal inter-hospital transport exposes critically ill and premature infants to a range of environmental stressors, including mechanical vibration, excessive noise, and temperature dysregulation, which may compound underlying pathophysiology and contribute to adverse outcomes. Despite growing recognition of this concern, the extent and clinical significance of these exposures remain incompletely characterised. The objective of this study is to systematically review the available literature on the physiological impact of environmental stressors during neonatal transport and synthesise evidence on biophysical exposures and clinical outcomes. A systematic search of MEDLINE, Embase, CINAHL, and the Cochrane Library was performed in accordance with the Preferred Reporting Items for Systematic Reviews and Meta-Analyses 2020 guidelines. The search covered the period from 1 January 2016 to 31 December 2025. Studies reporting environmental stressor measurements (vibration, noise, temperature, acceleration) and/or associated physiological outcomes in neonates or paediatric patients during inter-hospital transport were eligible. Risk of bias was assessed using the Newcastle-Ottawa Scale, the Joanna Briggs Institute critical appraisal checklist for quasi-experimental studies, and the AXIS tool, as appropriate. The review was not prospectively registered. Ten studies, published between 2016 and 2025, met the inclusion criteria. Whole-body vibration frequently approached or exceeded the European Union occupational action value (0.5 m/s²), reaching up to 70% of this value in individual transfers, although these adult-derived thresholds may not reflect true neonatal risk. Noise consistently surpassed the American Academy of Pediatrics-recommended ceiling of 45 dB(A). Hypothermia affected 8-40% of transported neonates, with the highest rates in very low birthweight infants under arctic conditions. Behavioural distress measures were significantly elevated throughout transfer procedures. Cerebral regional oxygen saturation drops of ≥20% from baseline occurred in 40-55% of transported patients in a single pilot study. Four studies were rated as low overall risk of bias, four as moderate, and two as high. Neonates undergoing inter-hospital transport are routinely exposed to environmental stressors at levels exceeding established adult safety thresholds, with consistent evidence of associated physiological and neurological risk. Several exposures, including vibration and thermal dysregulation, are potentially modifiable through routing optimisation, equipment design, and operational protocols. Neonatal-specific safety standards are urgently needed.

## Introduction and background

The centralisation of neonatal intensive care has made inter-hospital transport of critically ill and preterm infants an essential and increasingly common component of perinatal healthcare delivery across high-income countries [1]. In the United Kingdom alone, over 15,000 neonatal transfers are conducted annually, and comparable patterns of regionalisation prevail across Europe, North America, and Australasia [2]. Although the clinical rationale for transferring sick newborns to specialist centres is well established, mounting epidemiological evidence indicates that postnatal transport is independently associated with increased morbidity and mortality. Transported extremely preterm infants exhibit higher rates of severe intraventricular haemorrhage (IVH) compared with their inborn counterparts [3], and observational cohort studies have demonstrated elevated risks of mortality following ex utero transfer (transfer of an infant after birth, as distinct from in utero transfer of the fetus before delivery) that persist after adjustment for confounders [4]. These findings raise an important but incompletely resolved question: to what extent are the adverse outcomes associated with neonatal transport attributable to the environmental stressors encountered during the journey itself, rather than to the underlying severity of illness or quality of pre-transfer stabilisation?

Neonatal transport vehicles generate a complex constellation of environmental stressors that include sustained mechanical vibration, high-amplitude noise, acceleration and deceleration forces, and thermoregulatory challenges arising from ambient temperature extremes and procedural disruptions [5]. The physiological susceptibility of preterm and critically ill neonates to these stimuli is substantially greater than that of adults, owing to immature autonomic regulation, reduced thermoregulatory capacity, incomplete myelination, a fragile germinal matrix vasculature (the highly vascular, structurally immature region of the developing brain that is prone to haemorrhage in preterm infants), and dependence on medical equipment that itself may be affected by transport conditions [6]. Prior studies have established that vibration during neonatal ground transport frequently exceeds occupational exposure limits designed for healthy adult workers [5] and that noise levels within ambulances are significantly higher than those recommended for vulnerable perinatal populations [7]. However, the existing literature is fragmented, methodologically heterogeneous, and has rarely examined the direct physiological consequences of these exposures, such as changes in cerebral oxygenation, vital sign trajectories, or neurological injury, within the same framework.

Despite the clinical urgency of this problem, no universally accepted neonatal-specific safety standards for vibration, noise, or thermoregulatory thresholds during transport currently exist, and research recommendations derived from available studies rely almost exclusively on adult occupational health benchmarks. Furthermore, the degree to which these exposures are modifiable through operational interventions, such as route selection, speed management, incubator design, or driving behaviour, remains underexplored at scale. This systematic review was conducted to address these gaps by synthesising the available evidence on the nature, magnitude, and physiological impact of environmental stressors during neonatal inter-hospital transport. Specifically, we aimed to: (i) characterise the types and magnitudes of environmental stressors reported during neonatal transport; (ii) examine their association with clinical and biophysical outcomes; and (iii) identify modifiable factors that may reduce exposure and improve outcomes for this vulnerable population.

## Review

Methodology

Study Design

This systematic review was conducted and reported in accordance with the Preferred Reporting Items for Systematic Reviews and Meta-Analyses (PRISMA) 2020 guidelines [8]. A predefined protocol specifying objectives, eligibility criteria, search strategy, and analysis plan was prepared before the review. The protocol was not registered in a public registry such as PROSPERO; this is acknowledged as a limitation of the review.

Eligibility Criteria

Types of studies: All original research study designs were eligible, including prospective and retrospective observational studies, cohort studies, cross-sectional studies, experimental studies (including controlled bench or simulation studies), and pilot studies. Conference papers reporting original empirical data were eligible if sufficient methodological detail was available for quality assessment. Systematic reviews, narrative reviews, editorials, case reports, and studies reporting only simulated or modelled data without empirical transport measurements were excluded.

Types of participants: Studies were eligible if they included neonatal or paediatric patients (age <18 years, with the primary focus on neonates and preterm infants) undergoing inter-hospital or intra-hospital transport, or if they measured transport incubator performance under standardised conditions applicable to the neonatal transport context. Studies of exclusively adult populations were excluded.

Types of exposures: Eligible studies were required to measure or report at least one environmental stressor relevant to the transport environment, including but not limited to mechanical vibration (whole-body vibration or head acceleration); noise and sound levels; thermal conditions (ambient temperature, patient temperature, or thermoregulatory outcomes); acceleration or deceleration forces; or composite measures of transport-related physiological stress. Studies examining only pharmacological, staffing, or logistical aspects of transport without environmental measurement were excluded.

Types of outcome measures: Primary outcomes included: (i) quantitative measures of environmental stressor magnitude; (ii) clinical pathological outcomes attributable to transport stress; and (iii) validated surrogate measures of patient distress. Secondary outcomes included identification of modifiable risk factors and operational interventions.

Information Sources and Search Strategy

A systematic search of the following four electronic databases was conducted: MEDLINE (via PubMed), Embase, CINAHL (via EBSCO), and the Cochrane Central Register of Controlled Trials (CENTRAL). Searches covered the period from 1 January 2016 to 31 December 2025 and were last updated in December 2025. The search strategy combined Medical Subject Headings (MeSH) and free-text terms encompassing: (i) neonatal/infant/paediatric transport; (ii) environmental stressors (vibration, noise, acceleration, temperature, thermoregulation); and (iii) pathological or clinical outcomes. A representative MEDLINE (PubMed) search string is provided in the Appendices. A supplementary hand-search of the reference lists of all included studies and relevant review articles was performed.

Study Selection

**Figure 1:**
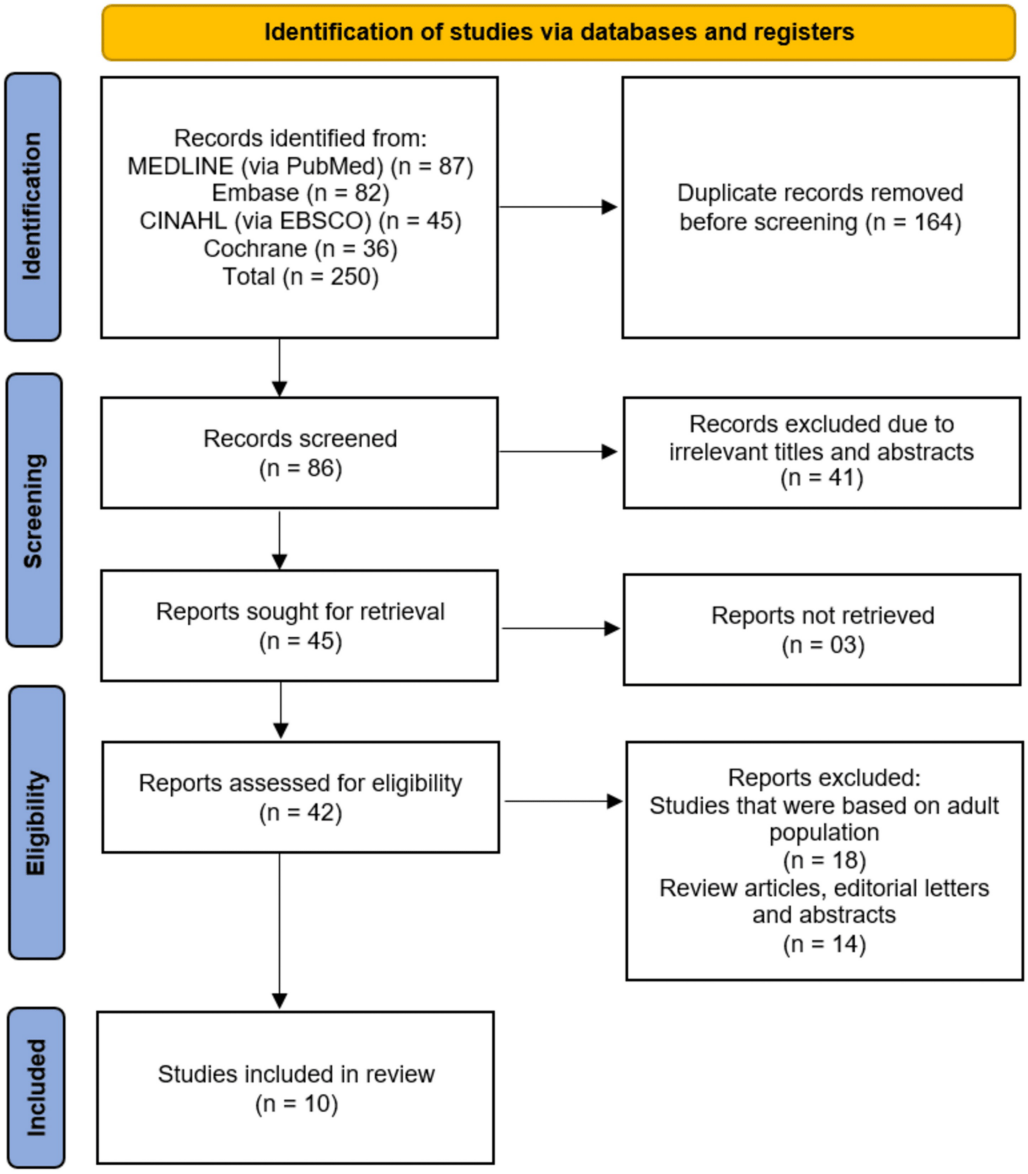
Preferred Reporting Items for Systematic Reviews and Meta-Analyses (PRISMA) flowchart illustrating the study selection process.

All identified citations were imported into EndNote X9 (Clarivate Analytics, Philadelphia, USA), which was used for automatic and manual removal of duplicates. Following deduplication, two independent reviewers screened all titles and abstracts against predefined eligibility criteria. Full-text articles were retrieved for all potentially eligible records and independently assessed by both reviewers. Disagreements at any stage were resolved by discussion and, where consensus could not be reached, by adjudication of a third reviewer. The screening process and reasons for exclusion at the full-text stage are documented in the Preferred Reporting Items for Systematic Reviews and Meta-Analyses (PRISMA) flow diagram (Figure 1).

Data Extraction

A standardised data extraction form was developed and pilot-tested on two included studies before full extraction. Two reviewers independently extracted data on study characteristics (design, setting, population, sample size), exposure measurement methods (instruments, sampling rates, calibration), stressors assessed, outcome measures, key quantitative results, and reported thresholds or reference standards. Discrepancies were resolved by consensus.

Quality Assessment and Risk of Bias

Risk of bias was assessed independently by two reviewers using study design-appropriate tools. Prospective and retrospective observational studies were appraised using the Newcastle-Ottawa Scale (NOS) [9], which evaluates the following three domains: selection of study groups, comparability of groups, and ascertainment of the outcome of interest. The Joanna Briggs Institute (JBI) Critical Appraisal Checklist for Quasi-Experimental Studies [10] was applied to the controlled bench experiment, which involved no human participants. The AXIS Tool for the Quality Assessment of Cross-Sectional Studies [11] was applied to the pilot and convenience-sampled feasibility studies. Overall risk of bias for each study was classified as low, moderate, or high based on the composite domain ratings. Disagreements were resolved by consensus.

Synthesis of Results

Due to substantial clinical and methodological heterogeneity across included studies, encompassing diverse study designs (observational cohorts, a controlled bench experiment, and feasibility/pilot studies), transport modalities (ground ambulance, helicopter, and fixed-wing aircraft), populations (term, preterm, and very low birthweight (VLBW) infants), outcome definitions, and measurement instruments and sampling rates, a formal meta-analysis was not appropriate. Studies were grouped by stressor type (vibration, noise, thermal, and acceleration) and by outcome domain (exposure magnitude, physiological/clinical outcome, and surrogate distress measure); where studies examined comparable outcomes, findings were compared against common reference benchmarks, and any discrepancies in extracted data were resolved by consensus between the two reviewers. Results were synthesised narratively. Qualitative synthesis followed a convergent integrated approach, grouping studies by stressor type and outcome domain.

Results

Study Selection

A total of 250 records were identified from MEDLINE, Embase, CINAHL, and Cochrane. After removing 164 duplicate records, 86 records were screened by title and abstract, leading to the exclusion of 41 irrelevant records. Of the remaining 45 reports sought for retrieval, 3 could not be obtained, leaving 42 reports assessed for full-text eligibility. At this stage, 18 reports were excluded because they were based on an adult population, and a further 14 were excluded as review articles, editorial letters, or abstracts. Consequently, 10 studies [12-21] met the inclusion criteria and were included in the review. The PRISMA flow diagram is presented in Figure 1.

Characteristics of Included Studies

The 10 included studies spanned 2016-2025 and were conducted across the following six countries: the United Kingdom (n = 3) [12,19,20], the United States (n = 3) [16,17,21], Norway (n = 1) [13], Japan (n = 1) [14], Hungary (n = 1) [15], and Switzerland (n = 1) [18]. Study designs comprised four prospective observational studies [12,15,19,20], one of which was reported as a conference paper [19], two retrospective cohort studies [16,17], one retrospective population-based audit [13], one controlled bench experiment [14], one prospective feasibility study [18], and one prospective pilot study [21]. Sample sizes ranged from 20 neonates [18] to 1,681 ambulance journeys [19]; denominators differed across studies, with some reporting individual infants (e.g., 620 neonates [16]; 109 infants [15]) and others reporting transport journeys (e.g., 1,487 journeys [12]; 882 acute transports [13]). Full study characteristics are presented in Table 1.

**Table 1:** Characteristics of the included studies. CICU: cardiac intensive care unit; GA: gestational age; HTB: head-to-back of vehicle; HTF: head-to-front of vehicle; IVH: intraventricular haemorrhage; NCH–LRI: Nottingham Children’s Hospital–Leicester Royal Infirmary; NETS-PCA: Neonatal Emergency and Transport Service of the Peter Cerny Foundation; NICU: neonatal intensive care unit; PMA: postmenstrual age; VLBW: very low birthweight

Author (year)	Study design	Setting	Population	N	Stressor(s) studied
Partridge et al. [12] (2025)	Prospective observational	CenTre Neonatal Transport, East Midlands, UK	Neonates in road ambulance transport	1,487 journeys (81,925 km)	Vibration; route type; road surface
Trulsen et al. [13] (2025)	Retrospective population-based audit	University Hospital of North Norway (Arctic); 30-year period (1994–2023)	Neonates undergoing air transport; VLBW sub-group (<1,500 g)	882 acute transports	Temperature control; glucose regulation; hypoglycaemia; IVH
Fukuyama and Arimitsu [14] (2023)	Controlled bench experiment	Keio University, Japan (laboratory)	No patients; ATOM IncuArch transport incubator at three ambient temperatures (20, 24, 28°C)	27 experimental runs	Thermoregulation; incubator access-port condition (open, covered, closed)
Lantos et al. [15] (2022)	Prospective observational	NETS-PCA, Budapest, Hungary	Mechanically ventilated neonates (term, preterm, extremely preterm); emergency inter-hospital transfers	109 infants	Vibration; frequency; sustained acceleration (speed/direction changes)
Greene et al. [16] (2022)	Retrospective cohort	Seattle Children’s Hospital regional network, USA; 2020–2021	Neonates <44 weeks PMA transported to a Level IV NICU or CICU	620 neonates	Composite transport stress (vibration, movement, altitude)
Sochet et al. [17] (2021)	Retrospective cohort	Johns Hopkins All Children’s Hospital, Florida, USA; 2016–2020	Children <18 years transported via fixed-wing aircraft in a subtropical climate	58 children (25 ≤5 kg; 33 >5 kg)	Environmental hyperthermia during fixed-wing transport
Zwissig et al. [18] (2019)	Prospective feasibility study	Lausanne University Hospital NICU, Switzerland	Clinically stable neonates transported by ground ambulance (non-emergency)	20 neonates	Vibration; movement; handling procedures
Partridge et al. [19] (2020)	Prospective observational (conference paper)	CenTre Neonatal Transport, NCH–LRI route, UK	Neonates in road ambulance transport	1,681 journeys (39 NCH–LRI)	Vibration; route choice/optimisation
Blaxter et al. [20] (2017)	Prospective observational	CenTre Neonatal Transport, UK; inter-hospital transfers	12 neonates (GA 24–41 weeks) + preterm manikin (three mattress types)	35 journeys (12 neonatal, 23 manikin)	Mechanical vibration; head acceleration
Valente et al. [21] (2016)	Prospective pilot study	Children’s Hospital Los Angeles, USA; 2011–2012	Paediatric/neonatal patients transported by ground, helicopter, or fixed-wing	24 patients	Z-axis peak acceleration; patient positioning (HTF vs. HTB)

The single controlled bench experiment (Fukuyama and Arimitsu [14]) was included because it provided direct, standardised measurement of transport-incubator thermoregulatory performance under conditions applicable to neonatal transport, addressing a mechanism (heat loss through incubator access ports) that cannot be feasibly or ethically isolated in live neonates. Because no infants were studied, its findings are interpreted as mechanistic and are considered separately from the clinical transport studies throughout the synthesis.

Mechanical Vibration Exposure

Five studies quantified mechanical (whole-body) vibration or peak axial acceleration during neonatal transport [12,15,19,20,21]. Whole-body vibration was assessed against ISO 2631-1, which remains the only internationally recognised standard for evaluating the human response to whole-body vibration [22]. Blaxter et al. [20] provided the most detailed frequency-domain characterisation in real patients, identifying a resonant frequency of approximately 9 Hz attributable to the mattress-harness system, which coupled with engine-derived chassis vibration peaking at 12-14 Hz. This resonance amplified vibration at the neonate’s head and torso by up to fourfold relative to the incubator chassis (3-5 dB for sponge and gel mattresses; 10 dB for the air mattress at 7 Hz). The cumulative A(8) vibration exposure reached at least 20% of the EU occupational action value (0.5 m/s²) in all 12 neonatal transports, and exceeded 70% of this value in two cases [20]. Direct head injury risk assessed by the Head Injury Criterion over a 15-millisecond window (HIC15) was negligible (maximum 9.3 vs. neonatal threshold ~99) [20]. Lantos et al. [15] confirmed that vibration, rather than speed changes or directional turns, was the dominant form of ambulance acceleration (median acceleration of 0.51 m/s² on the X-axis, with peaks exceeding 5 m/s²) and demonstrated that, while it did not significantly alter core ventilator parameters, intense vibration significantly increased the irregularity of pressure-volume (P-V) loops (median data pairs: 2,522 vs. 2,740; p < 0.0001). Partridge et al. [12] demonstrated that motorway routes produced significantly lower vibration than non-motorway alternatives (0.425 vs. 0.442 m/s²; p < 0.01), and that concrete road surfaces were associated with significantly greater vibration than asphalt (0.452 vs. 0.398 m/s²; p < 0.001).

Noise Exposure

Two studies quantified noise during neonatal transport [12,19]. Partridge et al. [12] reported that noise never fell below the American Academy of Pediatrics (AAP)-recommended ceiling of 45 dB(A) during any recorded transfer, with levels exceeding 70 dB(A) for over 60% of patient-journey time. A strong positive correlation between vehicle speed and noise was observed (r² = 0.91) [19]. Concrete road surfaces generated significantly higher noise levels than asphalt sections of the same road (77.8 vs. 76.1 dB(A); p < 0.001) [12]. In contrast to vibration, noise was not significantly reduced by motorway routing relative to non-motorway alternatives.

Thermoregulatory Challenges

Four included studies reported thermoregulatory outcomes [13,14,16,17]; three examined thermal exposure as a primary focus [13,14,17], while Greene et al. [16] captured temperature within a composite vital-sign analysis (described below). Trulsen et al. [13] documented hypothermia (<36.0°C) on neonatal intensive care unit arrival in 8.1% of all transported neonates across a 30-year period, rising to approximately 40% among outborn VLBW infants, markedly exceeding the 15% observed among inborn VLBW infants at the same centre. Despite three decades of equipment evolution, hypothermia rates in VLBW infants did not improve significantly between periods. Conversely, Sochet et al. [17] identified environmental hyperthermia as a distinct risk during fixed-wing transport in subtropical Florida: 36% of infants ≤5 kg experienced a ΔT ≥1°C compared with only 3% of those >5 kg (p < 0.01), with patient weight inversely associated with temperature elevation (odds ratio = 0.69). Fukuyama and Arimitsu [14] demonstrated mechanistically that opening incubator access ports during clinical procedures caused incubator temperatures to fall from 37.0°C to 33.5°C within 15 minutes at an ambient temperature of 20°C, while the use of port covers maintained temperatures equivalent to those of closed ports. These findings indicate that an inexpensive and immediately implementable intervention can meaningfully mitigate in-transit thermal loss.

Acceleration Forces and Cerebral Oxygenation

Valente et al. [21] quantified peak z-axis acceleration across three transport modalities, finding values of 0.05-0.20 g, small, comparable, and substantially lower than adult pilot exposures of 4-7 g. No statistically significant associations were identified between peak accelerations or patient positioning and regional oxygen saturation (rSO₂) drops during takeoff or landing. However, 40-55% of patients experienced rSO₂ drops ≥20% from baseline during the full course of transport, which is consistent with cumulative physiological exposure, rather than discrete acceleration events, being the more relevant mechanism.

Composite Physiological Trajectories and Behavioural Outcomes

Greene et al. [16] applied group-based trajectory modelling to 620 transported neonates, finding that 92% had at least one abnormal systolic blood pressure (SBP) measurement, 53% followed a ‘low and decreasing’ SBP trajectory, 36% a ‘high and increasing’ heart rate trajectory, and 56% had at least one abnormal temperature. Postmenstrual age ≤28 weeks was the strongest independent predictor of adverse trajectories across all four vital signs (adjusted relative risk of 2.4-4.2 for various trajectories). Zwissig et al. [18] demonstrated that behavioural distress scores (Comfort Behavior (CB) and Premature Infant Pain Profile-Revised (PIPP-R)) approximately doubled from baseline during transfer and did not return to resting values, with peak responses at incubator handling steps. Salivary cortisol, by contrast, showed no significant change, likely reflecting its poor sensitivity at low-to-moderate stress levels in the neonatal population (Table 2).

**Table 2:** Quantitative outcome data and comparison against clinical or regulatory reference thresholds. AAP: American Academy of Pediatrics; AIS: Abbreviated Injury Scale; dB(A): A-weighted decibels; EU: European Union; GA: gestational age; HIC15: Head Injury Criterion over a 15-ms window; HR: heart rate; IVH: intraventricular haemorrhage; ISO: International Organization for Standardization; MEMS: micro-electromechanical system; NIRS: near-infrared spectroscopy; OR: odds ratio; P-V: pressure-volume; rSO₂: regional cerebral oxygen saturation; SBP: systolic blood pressure; VLBW: very low birthweight; WBV: whole-body vibration

Author (year)	Stressor	Measurement tool	Key quantitative result	Clinical threshold/Reference standard	Exceeded reference threshold?
Partridge et al. [12] (2025)	Vibration	Smartphone accelerometer; ISO 2631-1	Mean vibration 0.42 m/s²; exceeded 0.5 m/s² on 15.2% of patient-journey time	EU occupational action value: 0.5 m/s² A(8)	Yes
Partridge et al. [12] (2025)	Noise	Smartphone microphone; dB(A)	Noise >70 dB(A) for 61.2% of patient-transfer time; never fell below 45 dB(A)	AAP recommended ceiling: 45 dB(A)	Yes
Trulsen et al. [13] (2025)	Hypothermia	Rectal temperature probe	Hypothermia on arrival: 8.1% overall; ~40% of outborn VLBW infants	Normal: 36.0–37.5°C	Yes
Trulsen et al. [13] (2025)	Neurological injury (IVH)	Cranial ultrasound; Papile classification	Severe IVH Grade III–IV: 20.7% outborn VLBW vs. 5.9% inborn VLBW	Inborn rate used as comparator; no transport-specific threshold	Yes
Fukuyama & Arimitsu [14] (2023)	Thermal loss (incubator)	Internal thermometer; 15-min observation	Open ports at 20°C ambient: internal temperature fell from 37.0 to 33.5°C. Covered ports: 37.1°C maintained	Target: 37°C; guideline ambient ≥25°C	Yes (open ports)
Lantos et al. [15] (2022)	Vibration	Mobile accelerometer 100 Hz; Butterworth filter	Median acceleration 0.51 m/s² (X-axis); peaks >5 m/s² in most recordings	<1 m/s² typical; peaks >5 m/s² considered significant	Yes
Lantos et al. [15] (2022)	Vibration effect on ventilation	Fabian+nCPAP data logger (125 Hz); P-V loops	P-V loop complexity: 2,522 vs. 2,740 data pairs at low vs. high vibration (p < 0.0001)	No established threshold; statistically significant increase in irregularity	Yes (P-V loops)
Greene et al. [16] (2022)	Systolic blood pressure	Electronic transport records; every 5–15 min	92% had ≥1 abnormal SBP; 53% in ‘low and decreasing’ trajectory	GA-adjusted norms	Yes
Greene et al. [16] (2022)	Heart rate	Electronic transport records	36% in ‘high and increasing’ trajectory; median HR 148 bpm; tachycardia >180 bpm	GA-adjusted norms	Yes
Greene et al. [16] (2022)	Temperature	Skin/axillary probes; electronic record	56% had ≥1 abnormal temperature; 15% in ‘low and flat’ hypothermic trajectory	Normal: 36.5–37.4°C	Yes
Zwissig et al. [18] (2019)	Behavioural stress	CB scale; PIPP-R scale	CB score doubled (8.8 → 17.6) at incubator transfer step; did not return to baseline	CB ≥17 = pain/discomfort requiring intervention	Yes
Zwissig et al. [18] (2019)	Physiological stress (cortisol)	Salivary cortisol; enzyme immunoassay	No significant change (0.222 vs. 0.259 µg/dL; p=0.663)	No neonatal reference range established	No
Sochet et al. [17] (2021)	Environmental hyperthermia	Continuous skin temperature (ZOLL monitor)	ΔT: ≤5 kg group 0.8°C vs. >5 kg 0.2°C (p<0.01); 36% of ≤5 kg had ΔT ≥1°C	ΔT ≥1°C = clinically significant elevation; >38°C = fever	Yes (≤5 kg)
Partridge et al. [19] (2020)	Vibration (route comparison)	Smartphone accelerometer; ISO-weighted m/s²	Optimal route: 0.43 vs. average 0.47 m/s² (10% reduction)	EU occupational action value: 0.5 m/s²	Near limit
Blaxter et al. [20] (2017)	Vibration (whole-body)	MEMS inertial sensors; ISO 2631-1 A(8)	A(8) ≥20% of EU action value in all 12 neonatal transports; up to 70% in 2 cases	EU WBV action value: 0.5 m/s² A(8)	Yes
Blaxter et al. [20] (2017)	Linear head acceleration	MEMS forehead sensor; HIC15 standard	Maximum HIC15 = 9.3 (neonatal); maximum 13.1 (manikin)	Scaled neonatal HIC15 threshold ~99 (20% AIS 3+ risk)	No
Valente et al. [21] (2016)	Z-axis peak acceleration	GP1 accelerometer (100 Hz); z-axis (spine-aligned)	Ground: 0.16/0.08 g; helicopter: 0.16/0.05 g; fixed-wing: 0.14/0.20 g (takeoff/landing)	No established neonatal threshold; adult pilot: 4–7 g	No
Valente et al. [21] (2016)	Cerebral oxygenation (rSO₂)	INVOS-5100C NIRS cerebral oximeter	rSO₂ drop ≥20% during full transport: 55% ground, 50% helicopter, 40% fixed-wing	Abnormal rSO₂: ≥20% drop from baseline or absolute value <40%	Yes

Risk of Bias

Risk of bias ratings are summarised in Table 3. Four studies were rated as low overall risk of bias [12,14,15,20]; four as moderate [13,16,17,19]; and two as high [18,21]. The most prevalent sources of methodological concern were confounding (a moderate or high degree in seven studies), selection bias (two high-risk studies relied on small convenience samples with limited generalisability), and detection bias (retrospective designs depending on clinical documentation). The bench study [14] received uniformly low ratings given its controlled experimental conditions, although the absence of a live neonate in the incubator constrains direct clinical extrapolation. The conference publication [19] carried moderate reporting bias inherent to the abbreviated peer-review format.

**Table 3:** Risk of bias assessment. AXIS: Appraisal tool for Cross-Sectional Studies; JBI: Joanna Briggs Institute; N/A: not applicable; NOS: Newcastle-Ottawa Scale

Authors (year)	Design/Tool	Selection bias	Performance bias	Detection bias	Attrition bias	Reporting bias	Overall
Partridge et al. [12] (2025)	Prospective observational (NOS)	Low	Low	Low	Low	Low	Low
Trulsen et al. [13] (2025)	Retrospective audit (NOS)	Moderate	Moderate	Moderate	Low	Low	Moderate
Fukuyama & Arimitsu [14] (2023)	Bench experiment (JBI)	Low	Low	Low	N/A	Low	Low
Lantos et al. [15] (2022)	Prospective observational (NOS)	Low	Low	Low	Low	Low	Low
Greene et al. [16] (2022)	Retrospective cohort (NOS)	Low	Moderate	Moderate	Low	Low	Moderate
Sochet et al. [17] (2021)	Retrospective cohort (NOS)	Moderate	Moderate	Moderate	Low	Moderate	Moderate
Zwissig et al. [18] (2019)	Prospective feasibility (AXIS)	High	Moderate	Low	Moderate	Low	High
Partridge et al. [19] (2020)	Prospective observational (NOS)	Moderate	Low	Low	Low	Moderate	Moderate
Blaxter et al. [20] (2017)	Prospective observational (NOS)	Low	Low	Low	Low	Low	Low
Valente et al. [21] (2016)	Prospective pilot (AXIS)	High	Moderate	Moderate	Low	Low	High

Discussion

This systematic review synthesises evidence from 10 studies published between 2016 and 2025 and demonstrates, with consistency across diverse settings and methodological approaches, that neonates undergoing inter-hospital transport are routinely exposed to environmental stressors, such as mechanical vibration, noise, thermal instability, and acceleration forces, at levels that exceed the available adult reference thresholds. Because the included studies were predominantly observational, the relationships described below should be interpreted as associations rather than causal effects; the available evidence cannot establish that transport stressors directly cause the adverse physiological or neurological outcomes reported. Taken together, these findings nonetheless provide a compelling basis for urgency in both the standardisation of neonatal transport safety metrics and the implementation of evidence-based mitigation strategies. Several themes emerge from the synthesis that warrant detailed interpretation in the context of the broader literature.

The magnitude of mechanical vibration recorded across included studies is a source of consistent concern. ISO 2631-1 whole-body vibration A(8) values reached up to 70% of the EU occupational action value (0.5 m/s²) in individual neonatal transfers [20], and the largest contemporary study reported whole-body vibration exceeding the EU occupational action value on 15.2% of patient-journey time [12]. These findings align closely with earlier landmark work by Campbell et al. [23], who identified high-amplitude vibration in neonatal transport vehicles as far back as the 1980s, suggesting that despite four decades of engineering advancement in vehicle design and incubator technology, the fundamental vibration hazard has remained incompletely addressed. A particularly important mechanistic finding from Blaxter et al. [20], that the resonant frequency of the mattress-harness system (~9 Hz) closely approximates the dominant engine-related chassis vibration peak (~12-14 Hz), explains why vibration is amplified rather than attenuated by the incubator suspension under typical transport conditions. This resonance-amplification mechanism was also documented by Gajendragadkar et al. [24], who found that sponge mattresses amplified vibration by factors of 2.2 to 3.4, and who concluded that replacement with gel mattresses offered only marginal benefit, a conclusion corroborated by Blaxter et al. [20]. These converging observations suggest that redesigning the incubator-vehicle coupling, for example, through active vibration-damping systems or pneumatic anti-vibration mounts, as employed by Lantos et al. [15], represents a more promising engineering solution than iterative mattress substitution.

The finding by Lantos et al. [15] that high vibration does not significantly alter macroscopic ventilator parameters is reassuring from a practical standpoint and supports the safe use of advanced ventilation modes, including synchronised and volume-targeted ventilation, during neonatal transport. However, the observation that intense vibration significantly increased the irregularity of P-V loops warrants further investigation. Irregular P-V loops indicate disrupted ventilator-patient synchrony, which may reflect direct physical interference with flow sensors or circuit elements, or alternatively physiological responses of the infant, including altered respiratory drive or muscle tone in response to vibration. Although no acute clinical deterioration was observed, subclinical ventilator-patient dyssynchrony during transport in extremely preterm infants could theoretically contribute to volume-pressure injury or aggravate germinal matrix instability. Future studies incorporating continuous respiratory function monitoring alongside accelerometry are needed to clarify this relationship.

Noise exposure data across the included studies uniformly demonstrate exceedance of the 45 dB(A) AAP ceiling, with average levels exceeding 70 dB(A) for the majority of patient-journey time [12,19]. This is substantially higher than the ambient noise levels of 50-65 dB(A) typically reported in occupied neonatal intensive care units [12], and raises specific concern for the hearing development of preterm infants, up to 10% of whom already experience hearing impairment compared with an estimated 0.1% in the general population [12]. Bouchut et al. [25] compared sound and vibration levels during helicopter and ground ambulance transport and found that helicopter transport was associated with higher noise but lower vibration, suggesting that the choice of transport mode entails trade-offs across stressor domains that cannot be resolved by a single optimisation criterion. The strong correlation between vehicle speed and noise identified in two studies [12,19] implies a structural tension between efficiency and acoustic comfort: faster transport reduces exposure duration but increases per-unit-time noise intensity. This trade-off, combined with the finding that motorway routing reduces vibration but not noise, underscores the necessity of a composite neonatal environmental comfort metric that weights multiple stressor dimensions simultaneously.

Thermoregulatory findings across the included studies highlight two physiologically opposite but equally important risks. The persistent high rates of hypothermia, affecting 8% of all transported neonates and approximately 40% of outborn VLBW infants in the 30-year Norwegian cohort [13], are consistent with data from multiple prior studies documenting that admission hypothermia is an independent predictor of mortality and morbidity in preterm neonates, with each 1°C reduction in admission temperature associated with a 28% increase in mortality among extremely low birthweight infants [13]. The mechanistic insight provided by Fukuyama and Arimitsu [14], that open incubator access ports cause internal temperatures to fall by as much as 3.5°C within 15 minutes, is clinically actionable and addresses a previously overlooked source of iatrogenic heat loss during procedures performed en route. The cost of access-port covers (approximately USD 2.25 per cover) renders this intervention immediately scalable without resource barriers. Conversely, Sochet et al. [17] identified a largely unreported risk of environmental hyperthermia during subtropical fixed-wing transport, with 36% of infants ≤5 kg experiencing clinically significant temperature elevations, a finding consistent with epidemiological data documenting disproportionate heat-related mortality in children under one year of age [17]. The divergence between hypothermia and hyperthermia risk across climatic contexts reinforces the importance of individualised environmental assessment rather than standardised thermal protocols, particularly as neonatal transport services expand into a wider range of geographical and seasonal conditions.

The vital sign trajectory analysis by Greene et al. [16] provides a uniquely longitudinal perspective on physiological stability during transport. The observation that 92% of transported neonates recorded at least one abnormal SBP measurement, and that over half followed a progressively worsening haemodynamic trajectory, is striking and suggests that haemodynamic instability during transport is far more prevalent than is captured by adverse-event reporting systems, which typically register only clinically recognised deteriorations. Karlsson et al. [26] similarly documented significant increases in heart rate variability during neonatal ground transport, proposing heart rate variability as a more sensitive physiological indicator of transport-related stress than mean heart rate alone. The very high-risk profile of infants ≤28 weeks PMA, at 2.4-4.2 times greater risk of adverse vital sign trajectories than term equivalents, is congruent with population-based outcome data demonstrating disproportionate adverse outcomes in extremely preterm outborn infants. These data collectively make a strong argument for enhanced continuous monitoring, targeted pre-transport stabilisation, and tailored transport protocols for the most premature infants.

The evidence on neurological outcomes, while limited in scope within this review, is among the most clinically significant. Trulsen et al. [13] documented a 3.5-fold higher rate of severe IVH (Grades III-IV) among outborn VLBW infants transported within the first 48 hours of life (20.7%) compared with inborn VLBW infants at the same centre (5.9%). This finding is consistent with the body of literature linking postnatal transport with IVH risk [3], including the large observational cohort study by Helenius et al. [4], which demonstrated, using propensity-score matching, that early postnatal transfer and birth outside a tertiary centre were independently associated with both mortality and severe brain injury in extremely preterm infants. The mechanistic pathways linking transport stressors to IVH remain incompletely understood but are hypothesised to include vibration-induced fluctuations in cerebral blood flow velocity, hypercapnia or hypocapnia from suboptimal ventilation, haemodynamic instability, and possibly the cumulative physiological burden of multiple stressors acting concurrently on a fragile germinal matrix vasculature. The finding by Valente et al. [21] that 40-55% of transported patients experienced rSO₂ drops of ≥20% from baseline during the transport period, without associated clinical deterioration, suggests that subclinical cerebral deoxygenation may be common and undetected under current monitoring practices, providing a plausible (though, given the observational design, unconfirmed) neurophysiological link to subsequent parenchymal injury. The routine incorporation of cerebral near-infrared spectroscopy monitoring during neonatal transport warrants serious consideration and prospective evaluation.

Evidence from the two route-optimisation studies [12,19] demonstrates clearly that vibration exposure is a modifiable determinant of transport quality, achievable without changes to vehicle specifications or clinical equipment. Optimised routing reduced mean vibration by 10% while simultaneously reducing journey time in the case study examined [19], challenging the common assumption that comfort and efficiency are in opposition. The smartphone-based data-collection approach used in both studies, validated against laboratory reference devices and deployable by clinical staff without engineering expertise, represents a significant methodological advance that enables large-scale, real-world measurement at a fraction of the cost of bespoke sensor systems. The collection of 946 million data points from 1,487 journeys [12] achieves a level of statistical power previously unavailable in neonatal transport research, and the demonstration of significant road-surface effects, with concrete producing meaningfully higher vibration and noise than asphalt at similar speeds, introduces a new and practical dimension to transport route planning. Future implementation studies should examine whether providing transport teams with live route-quality feedback via smartphone applications results in measurable reductions in exposure and improvements in patient outcomes.

Limitations

Several limitations of this systematic review and the underlying evidence base should be acknowledged. First, the review protocol was not prospectively registered, and the full multi-database search strategy was not published (only a representative MEDLINE string is provided in the Appendices), which may limit reproducibility. Second, the absence of neonatal-specific safety standards for vibration, noise, or thermal conditions during transport means that all threshold comparisons in the included studies rely on adult occupational health benchmarks; these thresholds were not developed for immature neonatal physiology, may not represent meaningful clinical risk thresholds for this population, and may either over- or under-estimate true physiological risk. Third, the methodological heterogeneity of included studies, spanning experimental bench studies, large-scale observational datasets, and small feasibility pilots, precluded formal meta-analysis and limits the precision of comparative synthesis. Fourth, most included studies were conducted in high-income settings with well-resourced transport services; findings may not be generalisable to low- and middle-income countries where transport conditions, incubator quality, and team expertise differ substantially. Fifth, several included studies [18,21] were rated as high overall risk of bias due to small convenience samples, which constrains confidence in their specific effect estimates. Sixth, no included study provided direct longitudinal data linking quantified transport stressor exposure to long-term neurodevelopmental outcomes, leaving the most clinically important causal question unanswered. Future prospective studies with individual-level stressor measurement, linked to neurodevelopmental follow-up, are essential to close this gap.

## Conclusions

Neonatal inter-hospital transport routinely exposes critically ill and preterm infants to mechanical vibration, noise, and thermal stressors at levels that exceed established adult safety thresholds, with associated physiological instability, neurological risk, and measurable behavioural distress. Several of these exposures, including vibration through route and surface selection, thermal loss through access-port cover use, and hyperthermia through pre-boarding aircraft cooling, are potentially modifiable through low-cost operational and engineering interventions. The development and adoption of neonatal-specific environmental safety standards for transport, informed by large-scale prospective measurement studies, represents an urgent research and policy priority. Investment in transport-equipment re-engineering, particularly vibration-dampening incubator coupling systems and validated thermoregulatory controls, and the routine use of cerebral near-infrared spectroscopy monitoring should be regarded as future research and development priorities requiring prospective evaluation, rather than as established, evidence-based standards of care. Until such standards and technologies are established, transport teams should apply existing evidence to minimise avoidable stressor exposures and prioritise pre-transfer stabilisation of the most vulnerable infants, particularly those born at extremely low gestational ages.

## Appendices

Representative MEDLINE (PubMed) search strategy

(neonatal* OR newborn* OR infant* OR preterm* OR premature OR “very low birth weight” OR VLBW) AND (transport* OR transfer* OR “inter-hospital” OR interhospital OR retrieval OR ambulance OR “fixed-wing” OR helicopter OR aeromedical) AND (vibration OR “whole-body vibration” OR noise OR “sound level” OR acceleration OR deceleration OR temperature OR thermoregulat* OR hypothermia OR hyperthermia OR “environmental stress*”) AND (physiolog* OR “cerebral oxygenation” OR “near-infrared spectroscopy” OR rSO2 OR “heart rate” OR “blood pressure” OR “intraventricular haemorrhage” OR IVH OR distress OR cortisol). Limits: 1 January 2016 to 31 December 2025; humans. Equivalent strategies, adapted to each interface, were run in Embase, CINAHL (EBSCO), and CENTRAL.
